# Supplementary material for: Discovery and application of insertion-deletion (INDEL) polymorphisms for QTL mapping of early life-history traits in Atlantic salmon
Source: BMC Genomics. 2010 Mar 8;11:156. doi: 10.1186/1471-2164-11-156 (PMC2838853; doi:10.1186/1471-2164-11-156)
Supplement: Additional file 2 — Information on developed 76 locus single-run INDEL panel in Atlantic salmon. Information on fluorescence labeling, primer concentrations, PCR pooling and links to alignments, INDEL motifs and GENESCAN (Burge and Karlin 1997) predictions of genes/exons are available in html format. [file 1471-2164-11-156-S2.ZIP › Additionalfile2/table8396.html]

Contig Alignment

```
Base	A B C D E F G H I J K L M N O P Q R S T U   
1	. . . . . . . . G . G . . . . . . . . . .    
2	. . . . . . . . G . G . . . . . . . . . .    
3	. . . . . . . . T . T . . . T . . . . . .    
4	G . G . . . . . G G G G . . G . . . . . .    
5	T . T . . . . . T T T T . . T . . . . . .    
6	G . G . . . . . G G G G . . G . . . . . .    
7	G . G . . . . . G G G G . . G . . . . . .    
8	A . A . . . . . A A A A . . A . . . . . .    
9	A . A . . . . . A A A A . . A . . . . . .    
10	A . A . . . . . A A A A . . A . . . . . .    
11	T . T . . . . . T T T T . . T . . . . . .    
12	C . C . . . . . C C C C . . C . . . . . .    
13	A . A . . . . . A A A A . . A . . . . . .    
14	C . C . . . . . C C C C . . C . . . . . .    
15	A . A . . . . . A A A A . . A . . . . . .    
16	A . A . . . . . A A A A . . A . . . . . .    
17	T . T . . . . . T T T T . . T . . . . . .    
18	T . T . . . . . T T T T . . T . . . . . .    
19	T . T . . . . . T T T T . . T . . . . . .    
20	A . A . . . . . A A A A . . A . . . . . .    
21	T . T . . . . . T T T T . . T . . . . . .    
22	T . T . . . . . T T T T . . T . . . . . .    
23	T . T . . . . . T T T T . . T . . . . . .    
24	C . C C . . . . C C C C . . C C . . . . .    
25	A . A A . . . . A A A A . . A A . . . . .    
26	T . T T . . . . T T T T . . T T . . . . .    
27	G . G G . . . . G G G G . . G G . . . . .    
28	A . A A . . . . A A A A . . A A . . . . .    
29	A . A A . . . . A A A A . . A A . . . . .    
30	A . A A . . . . A A A A . . A A . . . . .    
31	A . A A . . . . A A A A . . A A . . . . .    
32	T T T T . . . . T T T T . . T T . . . . .    
33	C C C C C . C C C C C C . . C C . . C . .    
34	A A A A A . A A A A A A . . A A . . A . .    
35	A A A A A . A A A A A A . . A A . . A . .    
36	A A A A A . A A A A A A . . A A . . A . .    
37	A A A A A . A A A A A A . . A A . . A . .    
38	T T T T T . T T T T T T . . T T . . T . .    
39	C C C C C . C C C C C C . . C C . . C . .    
40	A A A A A . A A A A A A . . A A . . A . .    
41	A A A A A . A A A A A A . . A A . . A . .    
42	C C C C C . C C C C C C . . C C . . C . .    
43	A A A A A . A A A A A A . . A A . . A . .    
44	C C C C C . C C C C C C . . C C . . C . .    
45	A A A A A . A A A A A A . . A A . . A . .    
46	G G G G G . G G G G G G . . G G . . G . .    
47	A A A A A . A A A A A A . . A A . . A . .    
48	A A A A A . A A A A A A . . A A . . A . .    
49	A A A A A . A A A A A A . . A A . . A . .    
50	C C C C C . C C C C C C . . C C . . C . .    
51	T T T T T . T T T T T T . . T T . . T . .    
52	G G G G G . G G G G G G . . G G . . G . .    
53	C C C C C . C C C C C C . . C C . . C . .    
54	A A A A A . A A A A A A . . A A . . A . .    
55	G G G G G . G G G G G G . . G G . . G . .    
56	C C C C C . C C C C C C . . C C . . C . .    
57	A A A A A . A A A A A A . . A A . . A . .    
58	A A A A A . A A A A A A . . A A . . A . .    
59	G G G G G . G G G G G G . . G G . . G . .    
60	G G G G G . G G G G G G . . G G . . G . .    
61	T T T T T . T T T T T T . . T T . . T . .    
62	A A A A A A A A A A A A A . A A . . A . .    
63	C C C C C C C C C C C C C . C C . . C . .    
64	A A A A A A A A A A A A A . A A . . A . .    
65	G G G G G G G G G G G G G . G G . . G . .    
66	T T T T T T T T T T T T T . T T . T T . .    
67	T T T T T T T T T T T T T . T T . T T . .    
68	T T T T T T T T T T T T T . T T . T T . .    
69	T T T T T T T T T T T T T . T T . T T . .    
70	C C C C C C C C C C C C C . C C . C C . .    
71	A A A A A A A A A A A A A . A A . A A . .    
72	A A A A A A A A A A A A A . A A . A A . .    
73	G G G G G G G G G G G G G . G G . G G . .    
74	T T T T T T T T T T T T T . T T . T T . .    
75	A A A A A A A A A A A A A . A A . A A . .    
76	T T T T T T T T T T T T T . T T . T T . .    
77	T T T T T T T T T T T T T . T T . T T . .    
78	C C C C C C C C C C C C C . C C . C C . .    
79	- - - A A A A A - - - - A . - A . A A . .  8	 indel  
80	- - - A A A A A - - - - A . - A . A A . .  8	 indel  
81	- - - T T T T T - - - - T . - T . T T . .  8	 indel  
82	- - - T T T T T - - - - T . - T . T T . .  8	 indel  
83	A A A A A A A A A A A A A . A A . A A . .    
84	T T T T T T T T T T T T T . T T . T T . .    
85	A A A A A A A A A A A A A . A A . A A . .    
86	G G G G G G G G G G G G G . G G . G G . .    
87	C C C C C C C C C C C C C . C C . C C . .    
88	A A A A A A A A A A A A A . A A . A A . .    
89	T T T T T T T T T T T T T . T T . T T . .    
90	T T T T T T T T T T T T T . T T . T T . .    
91	A A A A A A A A A A A A A . A A . A A . .    
92	A A A A A A A A A A A A A . A A . A A . .    
93	A A A A A A A A A A A A A . A A . A A . .    
94	A A A A A A A A A A A A A . A A . A A . .    
95	T T T T T T T T T T T T T . T T . T T . .    
96	C C C C C C C C C C C C C . C C . C C . .    
97	T T T T T T T T T T T T T . T T . T T . .    
98	A A A A A A A A A A A A A . A A . A A . .    
99	T T T T T T T T T T T T T . T T . T T . .    
100	G G G G G G G G G G G G G . G G . G G . .    
101	C C C C C C C C C C C C C . C C . C C . .    
102	A A A A A A A A A A A A A . A A . A A . .    
103	A A A A A A A A A A A A A . A A . A A . .    
104	T T T T T T T T T T T T T . T T . T T . .    
105	A A A A A A A A A A A A A . A A . A A . .    
106	C C C C C C C C C C C C C . C C . C C . .    
107	T T T T T T T T T T T T T . T T . T T . .    
108	C C C C C C C C C C C C C . C C . C C . .    
109	A A A A A A A A A A A A A . A A . A A . .    
110	A A A A A A A A A A A A A . A A . A A . .    
111	A A A A A A A A A A A A A . A A . A A . .    
112	T T T T T T T T T T T T T . T T . T T . .    
113	C C C C C C C C C C C C C . C C . C C . .    
114	C C C C C C C C C C C C C . C C . C C . .    
115	A A A A A A A A A A A A A . A A . A A . .    
116	. G G G G G G G G G G G G . G G . G G . .    
117	. A A A A A A A A A A A A . A A . A A . .    
118	. G G G G G G G G G G G G . G G . G G . .    
119	. T T T T T T T T T T T T . T T . T T . .    
120	. G G G G G G G G G G G G . G G . G G . .    
121	. G G G G G G G G G G G G . G G . G G . .    
122	. T T T T T T T T T T T T . T T . T T . .    
123	. T T T T T T T T T T T T . T T . T T . .    
124	. G G G G G G G G G G G G . G G . G G . .    
125	. G G G G G G G G G G G G . G G . G G . .    
126	. T T T T T T T T T T T T . T T . T T . .    
127	. A A A A A A A A A A A A . A A . A A . .    
128	. T T T T T T T T T T T T . T T . T T . .    
129	. G G G G G G G G G G G G . G G . G G . .    
130	. G G G G G G G G G G G G . G G . G G . .    
131	. A A A A A A A A A A A A . A A . A A . .    
132	. G G G G G G G G G G G G . G G . G G . .    
133	. G G G G G G G G G G G G . G G . G G . .    
134	. C C C C C C C C C C C C . C C . C C . .    
135	. A A A A A A A A A A A A . A A . A A . .    
136	. A A A A A A A A A A A A . A A . A A . .    
137	. G G G G G G G G G G G G . G G . G G . .    
138	. C C C C C C C C C C C C . C C . C C . .    
139	. A A A A A A A A A A A A . A A . A A . .    
140	. T T T T T T T T T T T T . T T . T T . .    
141	. T T T T T T T T T T T T . T T . T T . .    
142	. T T T T T T T T T T T T . T T . T T . .    
143	. C C C C C C C C C C C C . C C . C C . .    
144	. C C C C C C C C C C C C C C C . C C . .    
145	. C C C C C C C C C C C C C C C . C C . .    
146	. T T T T T T T T T T T T T T T . T T . .    
147	. C C C C C C C C C C C C C C C . C C . .    
148	. A A A A A A A A A A A A A A A . A A . .    
149	. T T T T T T T T T T T T T T T . T T . .    
150	. G G G G G G G G G G G G G G G . G G . .    
151	. G G G G G G G G G G G G G G G . G G . .    
152	. G G G G G G G G G G G G G G G . G G . .    
153	. T T T T T T T T T T T T T T T . T T . .    
154	. C C C C C C C C C C C C C C C . C C . .    
155	. C C C C C C C C C C C C C C C . C C . .    
156	. T T T T T T T T T T T T T T T . T T . .    
157	. T T T T T T T T T T T T T T T . T T . .    
158	. G G G G G G G G G G G G G G G . G G . .    
159	. G G G G G G G G G G G G G G G . G G . .    
160	. T T T T T T T T T T T T T T T . T T . .    
161	. T T T T T T T T T T T T T T T . T T . .    
162	. T T T T T T T T T T T T T T T . T T . .    
163	. T T T T T T T T T T T T T T T . T T . .    
164	. C C C C C C C C C C C C C C C . C C . .    
165	. A A A A A A A A A A A A A A A . A A . .    
166	. A A A A A A A A A A A A A A A . A A . .    
167	. G G G G G G G G G G G G G G G . G G . .    
168	. A A A A A A A A A A A A A A A . A A . .    
169	. C C C C C C C C C C C C C C C . C C . .    
170	. C C C C C C C C C C C C C C C . C C . .    
171	. A A A A A A A A A A A A A A A . A A . .    
172	. A A A A A A A A A A A A A A A . A A . .    
173	. G G G G G G G G G G G G G G G G G G . .    
174	. C C C C C C C C C C C C C C C C C C . .    
175	. A A A A A A A A A A A A A A A A A A . .    
176	. A A A A A A A A A A A A A A A A A A . .    
177	. T T T T T T T T T T T T T T T T T T . .    
178	. G G G G G G G G G G G G G G G G G G . .    
179	. T T T T T T T T T T T T T T T T T T . .    
180	. A A A A A A A A A A A A A A A A A A . .    
181	. A A A A A A A A A A A A A A A A A A . .    
182	. A A A A A A A A A A A A A A A A A A . .    
183	. G G G G G G G G G G G G G G G G G G . .    
184	. C C C C C C C C C C C C C C C C C C . .    
185	. A A A A A A A A A A A A A A A A A A . .    
186	. G G G G G G G G G G G G G G G G G G . .    
187	. G G G G G G G G G G G G G G G G G G . .    
188	. C C C C C C C C C C C C C C C C C C . .    
189	. A A A A A A A A A A A A A A A A A A . .    
190	. G G G G G G G G G G G G G G G G G G . .    
191	. A A A A A A A A A A A A A A A A A A . .    
192	. C C C C C C C C C C C C C C C C C C . .    
193	. C C C C C C C C C C C C C C C C C C . .    
194	. A A A A A A A A A A A A A A A A A A . .    
195	. C C C C C C C C C C C C C C C C C C . .    
196	. T T T T T T T T T T T T T T T T T T . .    
197	. T T T T T T T T T T T T T T T T T T T .    
198	. C C C C C C C C C C C C C C C C C C C .    
199	. A A A A A A A A A A A A A A A A A A A .    
200	. T T T T T T T T T T T T T T T T T T T .    
201	. A A A A A A A A A A A A A A A A A A A .    
202	. A A A A A A A A A A A A A A A A A A A .    
203	. G G G G G G G G G G G G G G G G G G G .    
204	. T T T T T T T T T T T T T T T T T T T .    
205	. A A A A A A A A A A A A A A A A A A A .    
206	. A A A A A A A A A A A A A A A A A A A .    
207	. G G G G G G G G G G G G G G G G G G G .    
208	. T T T T T T T T T T T T T T T T T T T .    
209	. A A A A A A A A A A A A A A A A A A A .    
210	. C C C C C C C C C C C C C C C C C C C .    
211	. A A A A A A A A A A A A A A A A A A A .    
212	. A A A A A A A A A A A A A A A A A A A .    
213	. G G G G G G G G G G G G G G G G G G G .    
214	. A A A A A A A A A A A A A A A A A A A .    
215	. A A A A A A A A A A A A A A A A A A A A    
216	. T T T T T T T T T T T T T T T T T T T T    
217	. C C C C C C C C C C C C C C C C C C C C    
218	. A A A A A A A A A A A A A A A A A A A A    
219	. T T T T T T T T T T T T T T T T T T T T    
220	. T T T T T T T T T T T T T T T T T T T T    
221	. T T T T T T T T T T T T T T T T T T T T    
222	. C C C C C C C C C C C C C C C C C C C C    
223	. A A A A A A A A A A A A A A A A A A A A    
224	. G G G G G G G G G G G G G G G G G G G G    
225	. T T T T T T T T T T T T T T T T T T T T    
226	. A A A A A G A A A A A A A A A A A A A G    
227	. T T T T T T T T T T T T T T T T T T T T    
228	. G G G G G G G G G G G G G G G G G G G G    
229	. A A A A A A A A A A A A A A A A A A A A    
230	. A A A A A A A A A A A A A A A A A A A A    
231	. A A A A A A A A A A A A A A A A A A A A    
232	. A A A A A A A A A A A A A A A A A A A A    
233	. T T T T T T T T T T T T T T T T T T T T    
234	. A A A A A A A A A A A A A A A A A A A A    
235	. C C C C C C C C C C C C C C C C C C C C    
236	. A A A A A A A A A A A A A A A A A A A A    
237	. T T T T T T T T T T T T T T T T T T T T    
238	. T T T T T T T T T T T T T T T T T T T T    
239	. C C C C C C C C C C C C C C C C C C C C    
240	. A A A A A A A A A A A A A A A A A A A A    
241	. A A A A A A A A A A A A A A A A A A A A    
242	. T T T T T T T T T T T T T T T T T T T T    
243	. A A A A A A A A A A A A A A A A A A A A    
244	. A A A A A A A A A A A A A A A A A A A A    
245	. T T T T T T T T T T T T T T T T T T T T    
246	. A A A A A A A A A A A A A A A A A A A A    
247	. A A A A A A A A A A A A A A A A A A A A    
248	. T T T T T T T T T T T T T T T T T T T T    
249	. A A A A A A A A A A A A A A A A A A A A    
250	. A A A A A A A A A A A A A A A A A A A A    
251	. T T T T T T T T T T T T T T T T T T T T    
252	. G G G G G G G G G G G G G G G G G G G G    
253	. C C C C C C C C C C C C C C C C C C C C    
254	. A A A A A A A A A A A A A A A A A A A A    
255	. A A A A A A A A A A A A A A A A A A A A    
256	. T T T T T T T T T T T T T T T T T T T T    
257	. C C C C C C C C C C C C C C C C C C C C    
258	. A A A A A A A A A A A A A A A A A A A A    
259	. T T T T T T T T T T T T T T T T T T T T    
260	. T T T T T T T T T T T T T T T T T T T T    
261	. C C C C C C C C C C C C C C C C C C C C    
262	. C C C C C C C C C C C C C C C C C C C C    
263	. A A A A A A A A A A A A A A A A A A A A    
264	. A A A A A A A A A A A A A A A A A A A A    
265	. A A A A A A A A A A A A A A A A A A A A    
266	. T T T T T T T T T T T T T T T T T T T T    
267	. G G G G G G G G G G G G G G G G G G G G    
268	. T T T T T T T T T T T T T T T T T T T T    
269	. A A A A A A A A A A A A A A A A A A A A    
270	. T T T T T T T T T T T T T T T T T T T T    
271	. C C C C C C C C C C C C C C C C C C C C    
272	. A A A A A A A A A A A A A A A A A A A A    
273	. A A A A A A A A A A A A A A A A A A A A    
274	. C C A A A A A C C C A A C A A A A A A A  6	 transversion  
275	. A A A A A A A A A A A A A A A A A A A A    
276	. G G G G G G G G G G G G G G G G G G G G    
277	. A A A A A A A A A A A A A A A A A A A A    
278	. A A A A A A A A A A A A A A A A A A A A    
279	. C C C C C C C C C C C C C C C C C C C C    
280	. T T T T T T T T T T T T T T T T T T T T    
281	. G G G G G G G G G G G G G G G G G G G G    
282	. A A A A A A A A A A A A A A A A A A A A    
283	. T T T T T T T T T T T T T T T T T T T T    
284	. T T T T T T T T T T T T T T T T T T T T    
285	. C C C C C C C C C C C C C C C C C C C C    
286	. T T T T T T T T T T T T T T T T T T T T    
287	. A A A A A A A A A A A A A A A A A A A A    
288	. T T T T T T T T T T T T T T T T T T T T    
289	. T T T T T T T T T T T T T T T T T T T T    
290	. T T T T T T T T T T T T T T T T T T T T    
291	. T T T T T T T T T T T T T T T T T T T T    
292	. C C C C C C C C C C C C C C C C C C C C    
293	. T T T T T T T T T T T T T T T T T T T T    
294	. G G G G G G G G G G G G G G G G G G G G    
295	. C C C C C C C C C C C C C C C C C C C C    
296	. T T T T T T T T T T T T T T T T T T T T    
297	. T T T T T T T T T T T T T T T T T T T T    
298	. A A A A A A A A A A A A A A A A A A A A    
299	. T T T T T T T T T T T T T T T T T T T T    
300	. G G G G G G G G G G G G G G G G G G G G    
301	. G G G G G G G G G G G G G G G G G G G G    
302	. T T T T T T T T T T T T T T T T T T T T    
303	. T T T T T T T T T T T T T T T T T T T T    
304	. A A A A A A A A A A A A A A A A A A A A    
305	. T T T T T T T T T T T T T T T T T T T T    
306	. G G G G G G G G G G G G G G G G G G G G    
307	. A A A A A A A A A A A A A A A A A A A A    
308	. A A A A A A A A A A A A A A A A A A A A    
309	. A A A A A A A A A A A A A A A A A A A A    
310	. A A A A A A A A A A A A A A A A A A A A    
311	. T T T T T T T T T T T T T T T T T T T T    
312	. C C C C C C C C C C C C C C C C C C C C    
313	. T T T T T T T T T T T T T T T T T T T T    
314	. A A A A A A A A A A A A A A A A A A A A    
315	. T T T T T T T T T T T T T T T T T T T T    
316	. G G G G G G G G G G G G G G G G G G G G    
317	. T T T T T T T T T T T T T T T T T T T T    
318	. C C C C C C C C C C C C C C C C C C C C    
319	. T T T T T T T T T T T T T T T T T T T T    
320	. A A A A A A A A A A A A A A A A A A A A    
321	. T T T T T T T T T T T T T T T T T T T T    
322	. A A A A A A A A A A A A A A A A A A A A    
323	. A A A A A A A A A A A A A A A A A A A A    
324	. C C C C C C C C C C C C C C C C C C C C    
325	. T T T T T T T T T T T T T T T T T T T T    
326	. G G G G G G G G G G G G G G G G G G G G    
327	. A A A A A A A A A A A A A A A A A A A A    
328	. T T T T T T T T T T T T T T T T T T T T    
329	. A A A A A A A A A A A A A A A A A A A A    
330	. T T T T T T T T T T T T T T T T T T T T    
331	. C C C C C C C C C C C C C C C C C C C C    
332	. T T T T T T T T T T T T T T T T T T T T    
333	. G G G G G G G G G G G G G G G G G G G G    
334	. A A A A A A A A A A A A A A A A A A A A    
335	. T T T T T T T T T T T T T T T T T T T T    
336	. T T T T T T T T T T T T T T T T T T T T    
337	. G G G G G G G G G G G G G G G G G G G G    
338	. T T T T T T T T T T T T T T T T T T T T    
339	. T T T T T T T T T T T T T T T T T T T T    
340	. T T T T T T T T T T T T T T T T T T T T    
341	. G G G G G G G G G G G G G G G G G G G G    
342	. T T T T T T T T T T T T T T T T T T T T    
343	. C C C C C C C C C C C C C C C C C C C C    
344	. T T T T T T T T T T T T T T T T T T T T    
345	. T T T T T T T T T T T T T T T T T T T T    
346	. T T T T T T T T T T T T T T T T T T T T    
347	. T T T T T T T T T T T T T T T T T T T T    
348	. A A A A A A A A A A A A A A A A A A A A    
349	. T T T T T T T T T T T T T T T T T T T T    
350	. C C C C C C C C C C C C C C C C C C C C    
351	. T T T T T T T T T T T T T T T T T T T T    
352	. G G G G G G G G G G G G G G G G G G G G    
353	. T T T T T T T T T T T T T T T T T T T T    
354	. T T T T T T T T T T T T T T T T T T T T    
355	. G G G G G G G G G G G G G G G G G G G G    
356	. C C C C C C C C C C C C C C C C C C C C    
357	. T T T T T T T T T T T T T T T T T T T T    
358	. T T T T T T T T T T T T T T T T T T T T    
359	. G G G G G G G G G G G G G G G G G G G G    
360	. G G G G G G G G G G G G G G G G G G G G    
361	. C C C C C C C C C C C C C C C C C C C C    
362	. A A A A A A A A A A A A A A A A A A A A    
363	. A A A A A A A A A A A A A A A A A A A A    
364	. T T T T T T T T T T T T T T T T T T T T    
365	. T T T T T T T T T T T T T T T T T T T T    
366	. A A A A A A A A A A A A A A A A A A A A    
367	. A A A A A A A A A A A A A A A A A A A A    
368	. A A A A A A A A A A A A A A A A A A A A    
369	. A A A A A A A A A A A A A A A A A A A A    
370	. T T T T T T T T T T T T T T T T T T T T    
371	. T C C C C C C C C C C C C C C C C C C C    
372	. . T T T T T T T T T T T T T T T T T T T    
373	. . G G G G G G G G G G G G G G G G G G G    
374	. . T T T T T T T T T T T T T T T T T T T    
375	. . T T T T T T T T T T T T T T T T T T T    
376	. . T T T T T T T T T T T T T T T T T T T    
377	. . T T T T T T T T T T T T T T T T T T T    
378	. . A A A A A A A A A A A A A A A A A A A    
379	. . A A A A A A A A A A A A A A A A A A A    
380	. . G G G G G G G G G G G G G G G G G G G    
381	. . A A A A A A A A A A A A A A A A A A A    
382	. . C C C C C C C C C C C C C C C C C C C    
383	. . A A A A A A A A A A A A A A A A A A A    
384	. . T T T T T T T T T T T T T T T T T T T    
385	. . A A A A A A A A A A A A A A A A A A A    
386	. . T T T T T T T T T T T T T T T T T T T    
387	. . G G G G G G G G G G G G G G G G G G G    
388	. . T T T T T T T T T T T T T T T T T T T    
389	. . T T T T T T T T T T T T T T T T T T T    
390	. . T T T T T T T T T T T T T T T T T T T    
391	. . C C C C C C C C C C C C C C C C C C C    
392	. . T T T T T T T T T T T T T T T T T T T    
393	. . A A A A A A A A A A A A A A A A A A A    
394	. . C C C C C C C C C C C C C C C C C C C    
395	. . A A A A A A A A A A A A A A A A A A A    
396	. . A A A A A A A A A A A A A A A A A A A    
397	. . A A A A A A A A A A A A A A A A A A A    
398	. . G G G G G G G G G G G G G G G G G G G    
399	. . A A A A A A A A A A A A A A A A A A A    
400	. . A A A A A A A A A A A A A A A A A A A    
401	. . A A A A A A A A A A A A A A A A A A A    
402	. . T T T T T T T T T T T T T T T T T T T    
403	. . A A A A A A A A A A A A A A A A A A A    
404	. . A A A A A A A A A A A A A A A A A A A    
405	. . A A A A A A A A A A A A A A A A A A A    
406	. . C C C C C C C C C C C C C C C C C C C    
407	. . A A A A A A A A A A A A A A A A A A A    
408	. . T T T T T T T T T T T T T T T T T T T    
409	. . G G G G G G G G G G G G G G G G G G G    
410	. . T T T T T T T T T T T T T T T T T T T    
411	. . A A A A A A A A A A A A A A A A A A A    
412	. . A A A A A A A A A A A A A A A A A A A    
413	. . A A A A A A A A A A A A A A A A A A A    
414	. . G G G G G G G G G G G G G G G G G G G    
415	. . T T T T T T T T T T T T T T T T T T T    
416	. . T T T T T T T T T T T T T T T T T T T    
417	. . T T T T T T T T T T T T T T T T T T T    
418	. . A A A A A A A A A A A A A A A A A A A    
419	. . A A A A A A A A A A A A A A A A A A A    
420	. . T T T T T T T T T T T T T T T T T T T    
421	. . C C C C C C C C C C C C C C C C C C C    
422	. . G G G G G G G G G G G G G G G G G G G    
423	. . T T T T T T T T T T T T T T T T T T T    
424	. . T T T T T T T T T T T T T T T T T T T    
425	. . T T T T T T T T T T T T T T T T T T T    
426	. . T T T T T T T T T T T T T T T T T T T    
427	. . T T T T T T T T T T T T T T T T T T T    
428	. . T T T T T T T T T T T T T T T T T T T    
429	. . C C C C C C C C C C C C C C C C C C C    
430	. . C C C C C C C C C C C C C C C C C C C    
431	. . C C C C C C C C C C C C C C C C C C C    
432	. . T T T T T T T T T T T T T T T T T T T    
433	. . G G G G G G G G G G G G G G G G G G G    
434	. . A A A A A A A A A A A A A A A A A A A    
435	. . C C C C C C C C C C C C C C C C C C C    
436	. . C C C C C C C C C C C C C C C C C C C    
437	. . A A A A A A A A A A A A A A A A A A A    
438	. . A A A A A A A A A A A A A A A A A A A    
439	. . T T T T T T T T T T T T T T T T T T T    
440	. . G G G G G G G G G G G G G G G G G G G    
441	. . T T T T T T T T T T T T T T T T T T T    
442	. . G G G G G G G G G G G G G G G G G G G    
443	. . C C C C C C C C C C C C C C C C C C C    
444	. . G G G G G G G G G G G G G G G G G G G    
445	. . C C C C C C C C C C C C C C C C C C C    
446	. . T T T T T T T T T T T T T T T T T T T    
447	. . G G G G G G G G G G G G G G G G G G G    
448	. . A A A A A A A A A A A A A A A A A A A    
449	. . A A A A A A A A A A A A A A A A A A A    
450	. . A A A A A A A A A A A A A A A A A A A    
451	. . A A A A A A A A A A A A A A A A A A A    
452	. . T T T T T T T T T T T T T T T T T T T    
453	. . G G G G G G G G G G G G G G G G G G G    
454	. . G G G G G G G G G G G G G G G G G G G    
455	. . A A A A A A A A A A A A A A A A A A A    
456	. . A A A A A A A A A A A A A A A A A A A    
457	. . A A A A A A A A A A A A A A A A A A A    
458	. . A A A A A A A A A A A A A A A A A A A    
459	. . T T T T T T T T T T T T T T T T T T T    
460	. . A A A A A A A A A A A A A A A A A A A    
461	. . C C C C C C C C C C C C C C C C C C C    
462	. . A A A A A A A A A A A A A A A A A A A    
463	. . T T T T T T T T T T T T T T T T T T T    
464	. . A A A A A A A A A A A A A A A A A A A    
465	. . C C C C C C C C C C C C C C C C C C C    
466	. . T T T T T T T T T T T T T T T T T T T    
467	. . A A A A A A A A A A A A A A A A A A A    
468	. . A A A A A A A A A A A A A A A A A A A    
469	. . C C C C C C C C C C C C C C C C C C C    
470	. . A A A A A A A A A A A A A A A A A A A    
471	. . A A A A A A A A A A A A A A A A A A A    
472	. . T T T T T T T T T T T T T T T T T T T    
473	. . A A A A A A A A A A A A A A A A A A A    
474	. . T T T T T T T T T T T T T T T T T T T    
475	. . A A A A A A A A A A A A A A A A A A A    
476	. . G G G G G G G G G G G G G G G G G G G    
477	. . G G G G G G G G G G G G G G G G G G G    
478	. . A A A A A A A A A A A A A A A A A A A    
479	. . G G G G G G G G G G G G G G G G G G G    
480	. . A A A A A A A A A A A A A A A A A A A    
481	. . G G G G G G G G G G G G G G G G G G G    
482	. . G G G G G G G G G G G G G G G G G G G    
483	. . G G G G G G G G G G G G G G G G G G G    
484	. . C C C C C C C C C C C C C C C C C C C    
485	. . T T T T T T T T T T T T T T T T T T T    
486	. . G G G G G G G G G G G G G G G G G G G    
487	. . A A A A A A A A A A A A A A A A A A A    
488	. . A A A A A A A A A A A A A A A A A A A    
489	. . G G G G G G G G G G G G G G G G G G G    
490	. . A A A A A A A A A A A A A A A A A A A    
491	. . T T T T T T T T T T T T T T T T T T T    
492	. . T T T T T T T T T T T T T T T T T T T    
493	. . G G G G G G G G G G G G G G G G G G G    
494	. . T T T T T T T T T T T T T T T T T T T    
495	. . A A A A A A A A A A A A A A A A A A A    
496	. . A A A A A A A A A A A A A A A A A A A    
497	. . A A A A A A A A A A A A A A A A A A A    
498	. . T T T T T T T T T T T T T T T T T T T    
499	. . G G G G G G G G G G G G G G G G G G G    
500	. . T T T T T T T T T T T T T T T T T T T    
501	. . T T T T T T T T T T T T T T T T T T T    
502	. . C C C C C C C C C C C C C C C C C C C    
503	. . G G G G G G G G G G G G G G G G G G G    
504	. . G G G G G G G G G G G G G G G G G G G    
505	. . T T T T T T T T T T T T T T T T T T T    
506	. . A A A A A A A A A A A A A A A A A A A    
507	. . G G G G G G G G G G G G G G G G G G G    
508	. . A A A A A A A A A A A A A A A A A A A    
509	. . A A A A A A A A A A A A A A A A A A A    
510	. . C C C C C C C C C C C C C C C C C C C    
511	. . T T T T T T T T T T T T T T T T T T T    
512	. . T T T T T T T T T T T T T T T T T T T    
513	. . T T T T T T T T T T T T T T T T T T T    
514	. . T T T T T T T T T T T T T T T T T T T    
515	. . C C C C C C C C C C C C C C C C C C C    
516	. . C C C C C C C C C C C C C C C C C C C    
517	. . T T T T T T T T T T T T T T T T T T T    
518	. . T T T T T T T T T T T T T T T T T T T    
519	. . C C C C C C C C C C C C C C C C C C C    
520	. . A A A A A A A A A A A A A A A A A A A    
521	. . C C C C C C C C C C C C C C C C C C C    
522	. . A A A A A A A A A A A A A A A A A A A    
523	. . A A A A A A A A A A A A A A A A A A A    
524	. . C C C C C C C C C C C C C C C C C C C    
525	. . A A A A A A A A A A A A A A A A A A A    
526	. . T T T T T T T T T T T T T T T T T T T    
527	. . G G G G G G G G G G G G G G G G G G G    
528	. . A A A A A A A A A A A A A A A A A A A    
529	. . G G G G G G G G G G G G G G G G G G G    
530	. . T T T T T T T T T T T T T T T T T T T    
531	. . T T T T T T T T T T T T T T T T T T T    
532	. . C C C C C C C C C C C C C C C C C C C    
533	. . T T T T T T T T T T T T T T T T T T T    
534	. . A A A A A A A A A A A A A A A A A A A    
535	. . T T T T T T T T T T T T T T T T T T T    
536	. . A A A A A A A A A A A A A A A A A A A    
537	. . A A A A A A A A A A A A A A A A A A A    
538	. . T T T T T T T T T T T T T T T T T T T    
539	. . G G G G G G G G G G G G G G G G G G G    
540	. . T T T G T T T T T T T T T T T T T T T    
541	. . G G G G G G G G G G G G G G G G G G G    
542	. . C C C C C C C C C C C C C C C C C C C    
543	. . T T T T T T T T T T T T T T T T T T T    
544	. . T T T T T T T T T T T T T T T T T T T    
545	. . A A A A A A A A A A A A A A A A A A A    
546	. . A A A A A A A A A A A A A A A A A A A    
547	. . C C C C C C C C C C C C C C C C C C C    
548	. . G G G G G G G G G G G G G G G G G G G    
549	. . A A A A A A A A A A A A A A A A A A A    
550	. . C C C C C C C C C C C C C C C C C C C    
551	. . C C C C C C C C C C C C C C C C C C C    
552	. . C A A A A A A A A A A A A A A A A A A    
553	. . . T T T T T T T T T T T T T T T T T T    
554	. . . A A A A A A A A A A A A A A A A A A    
555	. . . T T T T T T T T T T T T T T T T T T    
556	. . . G G G G G G G G G G G G G G G G G G    
557	. . . T T T T T T T T T T T T T T T T T T    
558	. . . A A A A A A A A A A A A A A A A A A    
559	. . . G G G G G G G G G G G G G G G G G G    
560	. . . T T T T T T T T T T T T T T T T T T    
561	. . . T T T T T T T T T T T T T T T T T T    
562	. . . A A A A A A A A A A A A A A A A A A    
563	. . . A A A A A A A A A A A A A A A A A A    
564	. . . G G G G G G G G G G G G G G G G G G    
565	. . . A A A A A A A A A A A A A A A A A A    
566	. . . T T T T T T T T T T T T T T T T T T    
567	. . . C C C C C C C C C C C C C C C C C C    
568	. . . T T T T T T T T T T T T T T T T T T    
569	. . . C C C C C C C C C C C C C C C C C C    
570	. . . T T T T T T T T T T T T T T T T T T    
571	. . . T T T T T T T T T T T T T T T T T T    
572	. . . A A A A A A A A A A A A A A A A A A    
573	. . . T T T T T T T T T T T T T T T T T T    
574	. . . G G G G G G G G G G G G G G G G G G    
575	. . . C C C C C C C C C C C C C C C C C C    
576	. . . C C C C C C C C C C C C C C C C C C    
577	. . . A A A A A A A A A A A A A A A A A A    
578	. . . T T T T T T T T T T T T T T T T T T    
579	. . . C C C C C C C C C C C C C C C C C C    
580	. . . A A A A A A A A A A A A A A A A A A    
581	. . . T T T T T T T T T T T T T T T T T T    
582	. . . A A A A A A A A A A A A A A A A A A    
583	. . . T T T T T T T T T T T T T T T T T T    
584	. . . T T T T T T T T T T T T T T T T T T    
585	. . . C C C C C C C C C C C C C C C C C C    
586	. . . A A A A A A A A A A A A A A A A A A    
587	. . . T T T T T T T T T T T T T T T T T T    
588	. . . A A A A A A A A A A A A A A A A A A    
589	. . . T T T T T T T T T T T T T T T T T T    
590	. . . T T T T T T T T T T T T T T T T T T    
591	. . . C C C C C C C C C C C C C C C C C C    
592	. . . A A A A A A A A A A A A A A A A A A    
593	. . . A A A A A A A A A A A A A A A A A A    
594	. . . T T T T T T T T T T T T T T T T T T    
595	. . . A A A A A A A A A A A A A A A A A A    
596	. . . T T T T T T T T T T T T T T T T T T    
597	. . . G G G G G G G G G G G G G G G G G G    
598	. . . T T T T T T T T T T T T T T T T T T    
599	. . . G G G G G G G G G G G G G G G G G G    
600	. . . A A A A A A A A A A A A A A A A A A    
601	. . . C C C C C C C C C C C C C C C C C C    
602	. . . A A A A A A A A A A A A A A A A A A    
603	. . . C C C C C C C C C C C C C C C C C C    
604	. . . T T T T T T T T T T T T T T T T T T    
605	. . . A A A A A A A A A A A A A A A A A A    
606	. . . A A A A A A A A A A A A A A A A A A    
607	. . . T T T T T T T T T T T T T T T T T T    
608	. . . C C C C C C C C C C C C C C C C C C    
609	. . . C C C C C C C C C C C C C C C C C C    
610	. . . A A A A A A A A A A A A A A A A A A    
611	. . . A A A A A A A A A A A A A A A A A A    
612	. . . A A A A A A A A A A A A A A A A A A    
613	. . . A A A A A A A A A A A A A A A A A A    
614	. . . A A A A A A A A A A A A A A A A A A    
615	. . . T A T T T T T T T T T T T T T T T T    
616	. . . . . A A A A A A A A A A A A A A A A    
617	. . . . . A A A A A A A A A A A A A A A A    
618	. . . . . T T T T T T T T T T T T T T T T    
619	. . . . . A A A A A A A A A A A A A A A A    
620	. . . . . A A A A A A A A A A A A A A A A    
621	. . . . . G G G G G G G G G G G G G G G G    
622	. . . . . C C C C C C C C C C C C C C C C    
623	. . . . . T T T T T T T T T T T T T T T T    
624	. . . . . T T T T T T T T T T T T T T T T    
625	. . . . . G G G G G G G G G G G G G G G G    
626	. . . . . A A A A A A A A A A A A A A A A    
627	. . . . . T T T T T T T T T T T T T T T T    
628	. . . . . G G G G G G G G G G G G G G G G    
629	. . . . . T T T T T T T T T T T T T T T T    
630	. . . . . T T T T T T T T T T T T T T T T    
631	. . . . . T T T T T T T T T T T T T T T T    
632	. . . . . C C C C C C C C C C C C C C C C    
633	. . . . . C C C C C C C C C C C C C C C C    
634	. . . . . T T T T T T T T T T T T T T T T    
635	. . . . . C C C C C C C C C C C C C C C C    
636	. . . . . T T T T T T T T T T T T T T T T    
637	. . . . . C C C C C C C C C C C C C C C C    
638	. . . . . T T T T T T T T T T T T T T T T    
639	. . . . . G G G G G G G G G G G G G G G G    
640	. . . . . G G G G G G G G G G G G G G G G    
641	. . . . . G T T T T T T T T T T T T T T T    
642	. . . . . . T T T T T T T T T T T T T T T    
643	. . . . . . T T T T T T T T T T T T T T T    
644	. . . . . . A A A A A A A A A A A A A A A    
645	. . . . . . C C C C C C C C C C C C C C C    
646	. . . . . . C C C C C C C C C C C C C C C    
647	. . . . . . A A A A A A A A A A A A A A A    
648	. . . . . . A A A A A A A A A A A A A A A    
649	. . . . . . T T T T T T T T T T T T T T T    
650	. . . . . . T T T T T T T T T T T T T T T    
651	. . . . . . C . C C C C C C C C C C C C C    
652	. . . . . . A . A A A A A A A A A A A A A    
653	. . . . . . G . G G G G G G G G G G G G G    
654	. . . . . . T . T T T T T T T T T T T T T    
655	. . . . . . G . G G G G G G G G G G G G G    
656	. . . . . . T . T T T T T T T T T T T T T    
657	. . . . . . C . C C C C C C C C C C C C C    
658	. . . . . . C . A A A A A A A A A A A A A    
659	. . . . . . . . T T T T T T T T T T T T T    
660	. . . . . . . . G G G G G G G G G G G G G    
661	. . . . . . . . A A A A A A A A A A A A A    
662	. . . . . . . . C C C C C C C C C C C C C    
663	. . . . . . . . C C C C C C C C C C C C C    
664	. . . . . . . . C C C C C C C C C C C C C    
665	. . . . . . . . C C C C C C C C C C C C C    
666	. . . . . . . . A A A A A A A A A A A A A    
667	. . . . . . . . T T T T T T T T T T T T T    
668	. . . . . . . . G G G G G G G G G G G G G    
669	. . . . . . . . T T T T T T T T T T T T T    
670	. . . . . . . . A A A A A A A A A A A A A    
671	. . . . . . . . A A A A A A A A A G A A A    
672	. . . . . . . . T T T T T T T T T T T T T    
673	. . . . . . . . T T T T T T T T T T T T T    
674	. . . . . . . . G G G G G G G G G G G G G    
675	. . . . . . . . G G G G G G G G G G G G G    
676	. . . . . . . . T T T T T T T T T T T T T    
677	. . . . . . . . T T T T T T T T T T T T T    
678	. . . . . . . . G G G G G G G G G G G G G    
679	. . . . . . . . A A A A A A A A A A A A A    
680	. . . . . . . . C C C C C C C C C C C C C    
681	. . . . . . . . C C C C C C C C C C C C C    
682	. . . . . . . . T T T T T T T T T T T T T    
683	. . . . . . . . G G G G G G G G G G G G G    
684	. . . . . . . . G . G G G G G G G G G G G    
685	. . . . . . . . A . A A A A A A A A A A A    
686	. . . . . . . . C . C C C C C C C C C C C    
687	. . . . . . . . A . A A A A A A A A A A A    
688	. . . . . . . . T . T T T T T T T T T T T    
689	. . . . . . . . T . T T T T T T T T T T T    
690	. . . . . . . . T . T T T T T T T T T T T    
691	. . . . . . . . T . T T T T T T T T T T T    
692	. . . . . . . . T . T T T T T T T T T T T    
693	. . . . . . . . C . C C C C C C C C C C C    
694	. . . . . . . . C . C T C C C C C C C C C    
695	. . . . . . . . C . C . C C C C C C C C C    
696	. . . . . . . . C . C . T T T T T T T T T    
697	. . . . . . . . . . . . G G G G G G G G G    
698	. . . . . . . . . . . . A A A A A A A A A    
699	. . . . . . . . . . . . C C C C C C C C C    
700	. . . . . . . . . . . . A A A A A A A A A    
701	. . . . . . . . . . . . G G G G G G G G G    
702	. . . . . . . . . . . . A A A A A A A A A    
703	. . . . . . . . . . . . A A A A A A A A A    
704	. . . . . . . . . . . . A A A A A A A A A    
705	. . . . . . . . . . . . T T T T T T T T T    
706	. . . . . . . . . . . . G G G G G G G G G    
707	. . . . . . . . . . . . T T T T T T T T T    
708	. . . . . . . . . . . . A A A A A A A A A    
709	. . . . . . . . . . . . G G G G G G G G G    
710	. . . . . . . . . . . . T T T T T T T T T    
711	. . . . . . . . . . . . C C C C C C C C C    
712	. . . . . . . . . . . . T T T T T T T T T    
713	. . . . . . . . . . . . T T T T T T T T T    
714	. . . . . . . . . . . . T T T T T T T T T    
715	. . . . . . . . . . . . T T T T T T T T T    
716	. . . . . . . . . . . . G G G G G G G G G    
717	. . . . . . . . . . . . T T T T T T T T T    
718	. . . . . . . . . . . . T T T T T T T T T    
719	. . . . . . . . . . . . A A A A A A A A A    
720	. . . . . . . . . . . . T T T T T T T T T    
721	. . . . . . . . . . . . C C C C C C C C C    
722	. . . . . . . . . . . . A A A A A A A A A    
723	. . . . . . . . . . . . C C C C C C C C C    
724	. . . . . . . . . . . . T T T T T T T T T    
725	. . . . . . . . . . . . C C C C C C C C C    
726	. . . . . . . . . . . . T T T T T T T T T    
727	. . . . . . . . . . . . G G G G G G G G G    
728	. . . . . . . . . . . . C C C C C C C C C    
729	. . . . . . . . . . . . A A A A A A A A A    
730	. . . . . . . . . . . . G G G G G G G G G    
731	. . . . . . . . . . . . T T T T T T T T T    
732	. . . . . . . . . . . . . G G G G G G G G    
733	. . . . . . . . . . . . . C C C C C C C C    
734	. . . . . . . . . . . . . G G G G G G G G    
735	. . . . . . . . . . . . . G G G G G G G G    
736	. . . . . . . . . . . . . C C C C C C C C    
737	. . . . . . . . . . . . . A A A A A A A A    
738	. . . . . . . . . . . . . G A A A A A A A    
739	. . . . . . . . . . . . . . A A A A A A A    
740	. . . . . . . . . . . . . . G G G G G G G    
741	. . . . . . . . . . . . . . T T T T T T T    
742	. . . . . . . . . . . . . . G G G G G G G    
743	. . . . . . . . . . . . . . C C C C C C C    
744	. . . . . . . . . . . . . . C C C C C C C    
745	. . . . . . . . . . . . . . A A A A A A A    
746	. . . . . . . . . . . . . . C C C C C C C    
747	. . . . . . . . . . . . . . A A A A A A A    
748	. . . . . . . . . . . . . . A A A A A A A    
749	. . . . . . . . . . . . . . G G G G G G G    
750	. . . . . . . . . . . . . . C C C C C C C    
751	. . . . . . . . . . . . . . A A A A A A A    
752	. . . . . . . . . . . . . . A A A A A A A    
753	. . . . . . . . . . . . . . A A A A A A A    
754	. . . . . . . . . . . . . . C C C C C C C    
755	. . . . . . . . . . . . . . A A A A A A A    
756	. . . . . . . . . . . . . . G G G G G G G    
757	. . . . . . . . . . . . . . A A A A A A A    
758	. . . . . . . . . . . . . . G G G G G G G    
759	. . . . . . . . . . . . . . C C C C C C C    
760	. . . . . . . . . . . . . . T T T T T T T    
761	. . . . . . . . . . . . . . G G G G G G G    
762	. . . . . . . . . . . . . . G G G G G G G    
763	. . . . . . . . . . . . . . G G G G G G G    
764	. . . . . . . . . . . . . . A A A A A A A    
765	. . . . . . . . . . . . . . C C C C C C C    
766	. . . . . . . . . . . . . . A A A A A A A    
767	. . . . . . . . . . . . . . G G G G G G G    
768	. . . . . . . . . . . . . . A A A A A A A    
769	. . . . . . . . . . . . . . T T T T T T T    
770	. . . . . . . . . . . . . . T T T T T T T    
771	. . . . . . . . . . . . . . T T T T T T T    
772	. . . . . . . . . . . . . . C C C C C C C    
773	. . . . . . . . . . . . . . A A A A A A A    
774	. . . . . . . . . . . . . . C C C C C C C    
775	. . . . . . . . . . . . . . A A A A A A A    
776	. . . . . . . . . . . . . . T T T T T T T    
777	. . . . . . . . . . . . . . G G G G G G G    
778	. . . . . . . . . . . . . . A A A A A A A    
779	. . . . . . . . . . . . . . T T T T T T T    
780	. . . . . . . . . . . . . . G G G G G G G    
781	. . . . . . . . . . . . . . G G G G G G G    
782	. . . . . . . . . . . . . . G G G G G G G    
783	. . . . . . . . . . . . . . T T T T T T T    
784	. . . . . . . . . . . . . . A A A A A A A    
785	. . . . . . . . . . . . . . A A A A A A A    
786	. . . . . . . . . . . . . . T T T T T T T    
787	. . . . . . . . . . . . . . T T T T T T T    
788	. . . . . . . . . . . . . . G G G G G G G    
789	. . . . . . . . . . . . . . G G G G G G G    
790	. . . . . . . . . . . . . . T T T T T T T    
791	. . . . . . . . . . . . . . T T T T T T T    
792	. . . . . . . . . . . . . . A A A A A A A    
793	. . . . . . . . . . . . . . G G G G G G G    
794	. . . . . . . . . . . . . . G G G G G G G    
795	. . . . . . . . . . . . . . G G G G G G G    
796	. . . . . . . . . . . . . . T T T T T T T    
797	. . . . . . . . . . . . . . G G G G G G G    
798	. . . . . . . . . . . . . . A A A A A A A    
799	. . . . . . . . . . . . . . G G G G G G G    
800	. . . . . . . . . . . . . . C C C C C C C    
801	. . . . . . . . . . . . . . A A A A A A A    
802	. . . . . . . . . . . . . . G G G G G G G    
803	. . . . . . . . . . . . . . C C C C C C C    
804	. . . . . . . . . . . . . . G G G G G G G    
805	. . . . . . . . . . . . . . T T T T T T T    
806	. . . . . . . . . . . . . . G G G G G G G    
807	. . . . . . . . . . . . . . C C C C C C C    
808	. . . . . . . . . . . . . . A A A A A A A    
809	. . . . . . . . . . . . . . A A A A A A A    
810	. . . . . . . . . . . . . . A A A A A A A    
811	. . . . . . . . . . . . . . G G G G G G G    
812	. . . . . . . . . . . . . . T T T T T T T    
813	. . . . . . . . . . . . . . T T T T T T T    
814	. . . . . . . . . . . . . . T T T T T T T    
815	. . . . . . . . . . . . . . C C C C C C C    
816	. . . . . . . . . . . . . . A A A A A A A    
817	. . . . . . . . . . . . . . C C C C C C C    
818	. . . . . . . . . . . . . . T T T T T T T    
819	. . . . . . . . . . . . . . A A A A A A A    
820	. . . . . . . . . . . . . . C C C C C C C    
821	. . . . . . . . . . . . . . C C C C C C C    
822	. . . . . . . . . . . . . . G G G G G G G    
823	. . . . . . . . . . . . . . T T T T T T T    
824	. . . . . . . . . . . . . . T T T T T T T    
825	. . . . . . . . . . . . . . C C C C C C C    
826	. . . . . . . . . . . . . . A A A A A A A    
827	. . . . . . . . . . . . . . A A A A A A A    
828	. . . . . . . . . . . . . . C A A A A A A    
829	. . . . . . . . . . . . . . . A A A A A A    
830	. . . . . . . . . . . . . . . G G G G G G    
831	. . . . . . . . . . . . . . . T T T T T T    
832	. . . . . . . . . . . . . . . T T T T T T    
833	. . . . . . . . . . . . . . . T T T T T T    
834	. . . . . . . . . . . . . . . G G G G G G    
835	. . . . . . . . . . . . . . . G G G G G G    
836	. . . . . . . . . . . . . . . G G G G G G    
837	. . . . . . . . . . . . . . . G G G G G G    
838	. . . . . . . . . . . . . . . T T T T T T    
839	. . . . . . . . . . . . . . . C C C C C C    
840	. . . . . . . . . . . . . . . A A A A A A    
841	. . . . . . . . . . . . . . . C C C C C C    
842	. . . . . . . . . . . . . . . C C C C C C    
843	. . . . . . . . . . . . . . . C C C C C T    
844	. . . . . . . . . . . . . . . A C A A A A    
845	. . . . . . . . . . . . . . . G . G G G G    
846	. . . . . . . . . . . . . . . A . A A A A    
847	. . . . . . . . . . . . . . . A . A A A A    
848	. . . . . . . . . . . . . . . A . A A A A    
849	. . . . . . . . . . . . . . . T . T T T T    
850	. . . . . . . . . . . . . . . G . G G G G    
851	. . . . . . . . . . . . . . . T . C T T T    
852	. . . . . . . . . . . . . . . C . . C C C    
853	. . . . . . . . . . . . . . . C . . C C C    
854	. . . . . . . . . . . . . . . T . . T T T    
855	. . . . . . . . . . . . . . . T . . T T T    
856	. . . . . . . . . . . . . . . G . . G G G    
857	. . . . . . . . . . . . . . . C . . C C T    
858	. . . . . . . . . . . . . . . C . . T C C    
859	. . . . . . . . . . . . . . . C . . . T C    
860	. . . . . . . . . . . . . . . . . . . . T    
861	. . . . . . . . . . . . . . . . . . . . C    
862	. . . . . . . . . . . . . . . . . . . . G    
863	. . . . . . . . . . . . . . . . . . . . A    
864	. . . . . . . . . . . . . . . . . . . . A    
865	. . . . . . . . . . . . . . . . . . . . A    
866	. . . . . . . . . . . . . . . . . . . . G    
867	. . . . . . . . . . . . . . . . . . . . A    
868	. . . . . . . . . . . . . . . . . . . . A    
869	. . . . . . . . . . . . . . . . . . . . A    
870	. . . . . . . . . . . . . . . . . . . . A    
871	. . . . . . . . . . . . . . . . . . . . G    
872	. . . . . . . . . . . . . . . . . . . . C    
873	. . . . . . . . . . . . . . . . . . . . A    
874	. . . . . . . . . . . . . . . . . . . . C    
875	. . . . . . . . . . . . . . . . . . . . A    
876	. . . . . . . . . . . . . . . . . . . . T    
877	. . . . . . . . . . . . . . . . . . . . T    
878	. . . . . . . . . . . . . . . . . . . . T    
879	. . . . . . . . . . . . . . . . . . . . T    
880	. . . . . . . . . . . . . . . . . . . . T    
881	. . . . . . . . . . . . . . . . . . . . T    
882	. . . . . . . . . . . . . . . . . . . . G    
883	. . . . . . . . . . . . . . . . . . . . T    
884	. . . . . . . . . . . . . . . . . . . . C    
885	. . . . . . . . . . . . . . . . . . . . C    
886	. . . . . . . . . . . . . . . . . . . . A    
887	. . . . . . . . . . . . . . . . . . . . C    
888	. . . . . . . . . . . . . . . . . . . . C    
889	. . . . . . . . . . . . . . . . . . . . A    
890	. . . . . . . . . . . . . . . . . . . . A    
891	. . . . . . . . . . . . . . . . . . . . A    
892	. . . . . . . . . . . . . . . . . . . . A    
893	. . . . . . . . . . . . . . . . . . . . T    
894	. . . . . . . . . . . . . . . . . . . . A    
895	. . . . . . . . . . . . . . . . . . . . A    
896	. . . . . . . . . . . . . . . . . . . . C    
897	. . . . . . . . . . . . . . . . . . . . A    
898	. . . . . . . . . . . . . . . . . . . . T    
899	. . . . . . . . . . . . . . . . . . . . C    
900	. . . . . . . . . . . . . . . . . . . . A    
901	. . . . . . . . . . . . . . . . . . . . A    
902	. . . . . . . . . . . . . . . . . . . . A    
903	. . . . . . . . . . . . . . . . . . . . T    
904	. . . . . . . . . . . . . . . . . . . . T    
905	. . . . . . . . . . . . . . . . . . . . G    
906	. . . . . . . . . . . . . . . . . . . . A    
907	. . . . . . . . . . . . . . . . . . . . T    
908	. . . . . . . . . . . . . . . . . . . . C    
909	. . . . . . . . . . . . . . . . . . . . A    
910	. . . . . . . . . . . . . . . . . . . . G    
911	. . . . . . . . . . . . . . . . . . . . A    
912	. . . . . . . . . . . . . . . . . . . . A    
913	. . . . . . . . . . . . . . . . . . . . A    
914	. . . . . . . . . . . . . . . . . . . . C    
915	. . . . . . . . . . . . . . . . . . . . A    
916	. . . . . . . . . . . . . . . . . . . . C    
917	. . . . . . . . . . . . . . . . . . . . A    
918	. . . . . . . . . . . . . . . . . . . . G    
919	. . . . . . . . . . . . . . . . . . . . T    
920	. . . . . . . . . . . . . . . . . . . . G    
921	. . . . . . . . . . . . . . . . . . . . T    
922	. . . . . . . . . . . . . . . . . . . . A    
923	. . . . . . . . . . . . . . . . . . . . G    
924	. . . . . . . . . . . . . . . . . . . . A    
925	. . . . . . . . . . . . . . . . . . . . C    
926	. . . . . . . . . . . . . . . . . . . . A    
927	. . . . . . . . . . . . . . . . . . . . T    
928	. . . . . . . . . . . . . . . . . . . . T    
929	. . . . . . . . . . . . . . . . . . . . G    
930	. . . . . . . . . . . . . . . . . . . . T    
931	. . . . . . . . . . . . . . . . . . . . T    
932	. . . . . . . . . . . . . . . . . . . . A    
933	. . . . . . . . . . . . . . . . . . . . A    
934	. . . . . . . . . . . . . . . . . . . . T    
935	. . . . . . . . . . . . . . . . . . . . G    
936	. . . . . . . . . . . . . . . . . . . . T    
937	. . . . . . . . . . . . . . . . . . . . T    
938	. . . . . . . . . . . . . . . . . . . . G    
939	. . . . . . . . . . . . . . . . . . . . C    
940	. . . . . . . . . . . . . . . . . . . . A    
941	. . . . . . . . . . . . . . . . . . . . A    
942	. . . . . . . . . . . . . . . . . . . . A    
943	. . . . . . . . . . . . . . . . . . . . C    
944	. . . . . . . . . . . . . . . . . . . . A    
945	. . . . . . . . . . . . . . . . . . . . A    
946	. . . . . . . . . . . . . . . . . . . . A    
947	. . . . . . . . . . . . . . . . . . . . A    
948	. . . . . . . . . . . . . . . . . . . . T    
949	. . . . . . . . . . . . . . . . . . . . A    
950	. . . . . . . . . . . . . . . . . . . . A    
951	. . . . . . . . . . . . . . . . . . . . A    
952	. . . . . . . . . . . . . . . . . . . . C    
953	. . . . . . . . . . . . . . . . . . . . T    
954	. . . . . . . . . . . . . . . . . . . . T    
955	. . . . . . . . . . . . . . . . . . . . G    
956	. . . . . . . . . . . . . . . . . . . . G    
957	. . . . . . . . . . . . . . . . . . . . A    
958	. . . . . . . . . . . . . . . . . . . . T    
959	. . . . . . . . . . . . . . . . . . . . T    
960	. . . . . . . . . . . . . . . . . . . . G    
961	. . . . . . . . . . . . . . . . . . . . G    
962	. . . . . . . . . . . . . . . . . . . . C    
963	. . . . . . . . . . . . . . . . . . . . T    
964	. . . . . . . . . . . . . . . . . . . . A    
965	. . . . . . . . . . . . . . . . . . . . A    
966	. . . . . . . . . . . . . . . . . . . . C    
967	. . . . . . . . . . . . . . . . . . . . A    
968	. . . . . . . . . . . . . . . . . . . . C    
969	. . . . . . . . . . . . . . . . . . . . A    
970	. . . . . . . . . . . . . . . . . . . . A    
971	. . . . . . . . . . . . . . . . . . . . C    
972	. . . . . . . . . . . . . . . . . . . . G    
973	. . . . . . . . . . . . . . . . . . . . T    
974	. . . . . . . . . . . . . . . . . . . . G    
975	. . . . . . . . . . . . . . . . . . . . C    
976	. . . . . . . . . . . . . . . . . . . . C    
977	. . . . . . . . . . . . . . . . . . . . A    
978	. . . . . . . . . . . . . . . . . . . . T    
979	. . . . . . . . . . . . . . . . . . . . T    
980	. . . . . . . . . . . . . . . . . . . . G    
981	. . . . . . . . . . . . . . . . . . . . G    
982	. . . . . . . . . . . . . . . . . . . . A    
983	. . . . . . . . . . . . . . . . . . . . A    
984	. . . . . . . . . . . . . . . . . . . . C    
985	. . . . . . . . . . . . . . . . . . . . A    
986	. . . . . . . . . . . . . . . . . . . . C    
987	. . . . . . . . . . . . . . . . . . . . A    
988	. . . . . . . . . . . . . . . . . . . . G    
989	. . . . . . . . . . . . . . . . . . . . G    
990	. . . . . . . . . . . . . . . . . . . . A    
991	. . . . . . . . . . . . . . . . . . . . G    
992	. . . . . . . . . . . . . . . . . . . . G    
993	. . . . . . . . . . . . . . . . . . . . G    
994	. . . . . . . . . . . . . . . . . . . . A    
995	. . . . . . . . . . . . . . . . . . . . T    
996	. . . . . . . . . . . . . . . . . . . . G    
997	. . . . . . . . . . . . . . . . . . . . G    
998	. . . . . . . . . . . . . . . . . . . . T    
999	. . . . . . . . . . . . . . . . . . . . T    
1000	. . . . . . . . . . . . . . . . . . . . G    
1001	. . . . . . . . . . . . . . . . . . . . C    
1002	. . . . . . . . . . . . . . . . . . . . T    
1003	. . . . . . . . . . . . . . . . . . . . G    
1004	. . . . . . . . . . . . . . . . . . . . A    
1005	. . . . . . . . . . . . . . . . . . . . T    
1006	. . . . . . . . . . . . . . . . . . . . A    
1007	. . . . . . . . . . . . . . . . . . . . A    
1008	. . . . . . . . . . . . . . . . . . . . T    
1009	. . . . . . . . . . . . . . . . . . . . T    
1010	. . . . . . . . . . . . . . . . . . . . G    
1011	. . . . . . . . . . . . . . . . . . . . G    
1012	. . . . . . . . . . . . . . . . . . . . C    
1013	. . . . . . . . . . . . . . . . . . . . C    
1014	. . . . . . . . . . . . . . . . . . . . T    
1015	. . . . . . . . . . . . . . . . . . . . C    
1016	. . . . . . . . . . . . . . . . . . . . T    
1017	. . . . . . . . . . . . . . . . . . . . G    
1018	. . . . . . . . . . . . . . . . . . . . T    
1019	. . . . . . . . . . . . . . . . . . . . A    
1020	. . . . . . . . . . . . . . . . . . . . C    
1021	. . . . . . . . . . . . . . . . . . . . G    
1022	. . . . . . . . . . . . . . . . . . . . G    
1023	. . . . . . . . . . . . . . . . . . . . T    
1024	. . . . . . . . . . . . . . . . . . . . T    
1025	. . . . . . . . . . . . . . . . . . . . A    
1026	. . . . . . . . . . . . . . . . . . . . T    
1027	. . . . . . . . . . . . . . . . . . . . G    
1028	. . . . . . . . . . . . . . . . . . . . T    
1029	. . . . . . . . . . . . . . . . . . . . A    
1030	. . . . . . . . . . . . . . . . . . . . G    
1031	. . . . . . . . . . . . . . . . . . . . A    
1032	. . . . . . . . . . . . . . . . . . . . T    
1033	. . . . . . . . . . . . . . . . . . . . A    
1034	. . . . . . . . . . . . . . . . . . . . T    
1035	. . . . . . . . . . . . . . . . . . . . T    
1036	. . . . . . . . . . . . . . . . . . . . C    
1037	. . . . . . . . . . . . . . . . . . . . C    
1038	. . . . . . . . . . . . . . . . . . . . G    
1039	. . . . . . . . . . . . . . . . . . . . T    
1040	. . . . . . . . . . . . . . . . . . . . A    
1041	. . . . . . . . . . . . . . . . . . . . A    
1042	. . . . . . . . . . . . . . . . . . . . A    
1043	. . . . . . . . . . . . . . . . . . . . A    
1044	. . . . . . . . . . . . . . . . . . . . A    
1045	. . . . . . . . . . . . . . . . . . . . A    
1046	. . . . . . . . . . . . . . . . . . . . T    
1047	. . . . . . . . . . . . . . . . . . . . C    
1048	. . . . . . . . . . . . . . . . . . . . T    
1049	. . . . . . . . . . . . . . . . . . . . G    
1050	. . . . . . . . . . . . . . . . . . . . C    
1051	. . . . . . . . . . . . . . . . . . . . C    
1052	. . . . . . . . . . . . . . . . . . . . G    
1053	. . . . . . . . . . . . . . . . . . . . T    
1054	. . . . . . . . . . . . . . . . . . . . T    
1055	. . . . . . . . . . . . . . . . . . . . T    
1056	. . . . . . . . . . . . . . . . . . . . C    
1057	. . . . . . . . . . . . . . . . . . . . C    
1058	. . . . . . . . . . . . . . . . . . . . A    
1059	. . . . . . . . . . . . . . . . . . . . G    
1060	. . . . . . . . . . . . . . . . . . . . C    
1061	. . . . . . . . . . . . . . . . . . . . T    
1062	. . . . . . . . . . . . . . . . . . . . A    
1063	. . . . . . . . . . . . . . . . . . . . C    
1064	. . . . . . . . . . . . . . . . . . . . C    
1065	. . . . . . . . . . . . . . . . . . . . C    
1066	. . . . . . . . . . . . . . . . . . . . C    
1067	. . . . . . . . . . . . . . . . . . . . C
```
